# Buddhism and Theosophy

**Published:** 1889-02

**Authors:** 


### HALL'S JOURNAL OF HEALTH

TRUTH DEMANDS NO SACRIFICE; ERROR CAN MAKE NONE.

VOL. 36 FEBRUARY, 1889. No. 2.

### BUDDHISM AND THEOSOPHY.

We publish in this number a very timely and much needed protest by Prof. Buchanan against the propagation of Buddhism and Buddhistic speculation in the United States under the deceptive name of Theosophy.

To those who know little or nothing of true Theosophy, the claims of the Theosophic Societies are very plausible and misleading. But those who are really masters in Theosophic Science are not to be gathered into an Oriental church which is wholly lacking in the spiritual element. The true home of Theosophy is in America where it is entirely untrammelled by any form of superstition. Orientalism has really done nothing for progress. Its influence is like that of the fumes of opium which make the victim insensible to the realities of life, a devotee to the dreams of Nirvana and an eternal past career of which there is not the slightest evidence

In all the twenty centuries gone by, what has Orientalism done to enlighten mankind in comparison with what America has done in less than half a century. Has it ever made a single scientific demonstration of the intercommunion of the two worlds? Not at all. That was achieved by Prof. Hare, while the so-called philosophers of India only muddled the subject and left their votaries uncertain of what existed beyond the confines of matter in what their fancy converted from a real world into a dreamy phantasmagoria.

Did the so-called philosophers of India ever reveal in a scientific form the grand laws of PSYCHOMETRY, the science which opens all realms of nature to the grasp of human intuition? No! their labors extended only toward a land of dreams. We are indebted to our countryman, Prof. BUCHANAN for this masterful science which enlarges the boundaries of all sciences, enlightens all religions and dissipates all superstition.

With all the sensitive and intuitional endowments of India at their command, did they ever throw any light upon the greatest of all sciences —the science of man? Did they ever give a single glimpse of a true Anthropology, or even of a true Physiology? Far from it. Their powers were wasted in the idle speculations of superstition, and the more one reads their lucubrations, the more ignorant he becomes of everything scientific and valuable in the department of Anthropology. This all comprehensive science is the fruit of the life-long labors of Prof. Buchanan, and constitutes in itself a system of Theosophy, which shows the relations of man to the supernal as well as the terrestrial world.

But from all this panorama of science and all the well-established facts of spiritualism, the Theosophic Society turns away to bury itself in the mysticism of a barbarous age, reminding us by its attitude, of the ostrich, endeavoring to elude the pursuer by thrusting its head in the sands of the desert.

